# Issues With Variability in Electronic Health Record Data About Race and Ethnicity: Descriptive Analysis of the National COVID Cohort Collaborative Data Enclave

**DOI:** 10.2196/39235

**Published:** 2022-09-06

**Authors:** Lily Cook, Juan Espinoza, Nicole G Weiskopf, Nisha Mathews, David A Dorr, Kelly L Gonzales, Adam Wilcox, Charisse Madlock-Brown, N3C Consortium

**Affiliations:** 1 Department of Medical Informatics and Clinical Epidemiology School of Medicine Oregon Health & Science University Portland, OR United States; 2 Department of Pediatrics Children’s Hospital Los Angeles Los Angeles, CA United States; 3 College of Human Sciences and Humanities University of Houston Clear Lake-Pearland, TX United States; 4 Citizen of the Cherokee Nation Portland, OR United States; 5 Joint School of Public Health Oregon Health & Science University-Portland State University Portland, OR United States; 6 Founding Indigenous Member BIPOC Decolonizing Data Council Portland, OR United States; 7 Indigenous Equity Institute Portland, OR United States; 8 Department of Medicine Institute for Informatics Washington University in St. Louis St. Louis, MO United States; 9 Tennessee Clinical and Translational Science Institute University of Tennessee Health Science Center Memphis, TN United States; 10 See Acknowledgments

**Keywords:** social determinants of health, health equity, bias, data quality, data harmonization, data standards, terminology, data aggregation

## Abstract

**Background:**

The adverse impact of COVID-19 on marginalized and under-resourced communities of color has highlighted the need for accurate, comprehensive race and ethnicity data. However, a significant technical challenge related to integrating race and ethnicity data in large, consolidated databases is the lack of consistency in how data about race and ethnicity are collected and structured by health care organizations.

**Objective:**

This study aims to evaluate and describe variations in how health care systems collect and report information about the race and ethnicity of their patients and to assess how well these data are integrated when aggregated into a large clinical database.

**Methods:**

At the time of our analysis, the National COVID Cohort Collaborative (N3C) Data Enclave contained records from 6.5 million patients contributed by 56 health care institutions. We quantified the variability in the harmonized race and ethnicity data in the N3C Data Enclave by analyzing the conformance to health care standards for such data. We conducted a descriptive analysis by comparing the harmonized data available for research purposes in the database to the original source data contributed by health care institutions. To make the comparison, we tabulated the original source codes, enumerating how many patients had been reported with each encoded value and how many distinct ways each category was reported. The nonconforming data were also cross tabulated by 3 factors: patient ethnicity, the number of data partners using each code, and which data models utilized those particular encodings. For the nonconforming data, we used an inductive approach to sort the source encodings into categories. For example, values such as “Declined” were grouped with “Refused,” and “Multiple Race” was grouped with “Two or more races” and “Multiracial.”

**Results:**

“No matching concept” was the second largest harmonized concept used by the N3C to describe the race of patients in their database. In addition, 20.7% of the race data did not conform to the standard; the largest category was data that were missing. Hispanic or Latino patients were overrepresented in the nonconforming racial data, and data from American Indian or Alaska Native patients were obscured. Although only a small proportion of the source data had not been mapped to the correct concepts (0.6%), Black or African American and Hispanic/Latino patients were overrepresented in this category.

**Conclusions:**

Differences in how race and ethnicity data are conceptualized and encoded by health care institutions can affect the quality of the data in aggregated clinical databases. The impact of data quality issues in the N3C Data Enclave was not equal across all races and ethnicities, which has the potential to introduce bias in analyses and conclusions drawn from these data. Transparency about how data have been transformed can help users make accurate analyses and inferences and eventually better guide clinical care and public policy.

## Introduction

The United States has had more COVID-19 cases and deaths than any other country [1]. Black or African American, Hispanic or Latino, and American Indian or Alaska Native (AI/AN) communities have experienced disproportionate morbidity and mortality from COVID-19 [2-5]. Compared with the non-Hispanic White population, the Black or African American population has a higher prevalence of COVID-19, as well as higher mortality and hospitalization rates from the virus [2]. The Centers for Disease Control and Prevention (CDC) reported that, between February 2020 and May 2020, Hispanic or Latino and non-White individuals under 65 years of age were 2 to 3 times more likely to die from COVID-19 than their non-Hispanic White counterparts [4]. COVID-19 incidence for AI/AN persons is estimated to be 3.5 times higher than for non-Hispanic White persons [5]. The full consideration of the social, economic, and health impacts of COVID-19 on these communities relies on data sets structured to answer such questions.

Resources have been created with the intention of tracking, quantifying, and analyzing the impact of COVID-19 within and across populations [6-8]. The largest such resource in the United States is the National COVID Cohort Collaborative (N3C), a National Institutes of Health (NIH)–funded collaboration between the National Center for Advancing Translational Sciences (NCATS) and the Center for Data to Health [8]. The N3C Data Enclave is also one of the largest collections of COVID-19 patient-level data globally [7], providing harmonized electronic health record (EHR) data from 56 health care institutions and networks across the country. Currently, 1615 researchers representing 186 research institutions have been granted access to the Enclave to work on 215 research projects [9].

Large data sets like N3C, whether centralized or distributed, face a substantial challenge in the form of data heterogeneity, stemming from varying data collection, documentation, and coding practices [10]. These upstream processes may result in data quality problems and other artifacts that can lead to data loss and possibly misleading signals in the data [11]. The encodings used to represent race and ethnicity vary across institutions and data models and require specialized harmonization [12]. Indeed, a significant technical challenge related to integrating race and ethnicity data across EHR systems is the lack of consistency in how data about race and ethnicity are collected and structured by health care organizations. The Institute of Medicine’s landmark report on racial and ethnic disparities in health care, *Unequal Treatment: Confronting Racial and Ethnic Disparities in Healthcare*, highlighted the need for standardized collection and reporting of race and ethnicity data [13].

Data standardization and harmonization is one of the best tools for combating heterogeneity and ensuring that observed signals are genuine. The N3C provides a unique opportunity to assess how different health care systems in various locations collect and conceptualize information about their patients’ race and ethnicity and to examine efforts to integrate these categories across different data models. In this paper, we discuss race and ethnicity from the perspective of data standards and database harmonization.

The standard most commonly used by health care systems to collect and organize data about race and ethnicity was created for the 2000 US Census. The Office of Management and Budget (OMB) released this standard in 1997 [14], and shortly afterward, the CDC added encodings to the OMB Standard; both are shown in Table 1 [15]. To maintain clarity and consistency, we used these terms throughout this paper.

The 1997 OMB classification system was then adopted with minor changes by Health Level Seven International (HL7), the creator of the standard most widely used by health care systems to transmit and receive health records [16]; any references to “the health care standard” in our paper refer to how this information is currently structured in HL7 Fast Healthcare Interoperability Resources (FHIR).

The current health care standard uses terminology in a manner different from how it is used colloquially. For the purposes of collecting and organizing self-reported patient demographic data, race and ethnicity are considered distinct concepts, and ethnicity refers only to Hispanic or Latino origin. Thus, ethnicity has 3 minimum codes: Patients can either be Hispanic or Latino or non-Hispanic or Latino. However, this category is intended to be hierarchical, and “granular” ethnicity refers to the 41 subcategories (e.g., Panamanian, Venezuelan) that are required to roll up into Hispanic or Latino.

**Table 1:** Office of Management and Budget (OMB) revisions to the Standards for the Classification of Federal Data on Race and Ethnicity, 1997.

OMB category	HL7^a^ code	Category definition
Race: American Indian or Alaska Native	1002-5	A person having origins in any of the original peoples of North and South America (including Central America) and who maintains tribal affiliation or community attachment
Race: Asian	2028-9	A person having origins in any of the original peoples of the Far East, Southeast Asia, or the Indian subcontinent including, for example, Cambodia, China, India, Japan, Korea, Malaysia, Pakistan, the Philippine Islands, Thailand, and Vietnam
Race: Black or African American	2054-5	A person having origins in any of the black racial groups of Africa. Terms such as “Haitian”; or “Negro”; can be used in addition to “Black or African American”
Race: Native Hawaiian or Other Pacific Islander	2076-8	A person having origins in any of the original peoples of Hawaii, Guam, Samoa, or other Pacific Islands
Race: White	2106-3	A person having origins in any of the original peoples of Europe, the Middle East, or North Africa
Ethnicity: Hispanic or Latino	2135-2	A person of Mexican, Puerto Rican, Cuban, South or Central American, or other Spanish culture or origin, regardless of race. Ethnicity is considered a distinct category from race

^a^HL7: Health Level Seven International.

The 2009 Institute of Medicine Subcommittee on Standardized Collection of Race/Ethnicity Data for Healthcare Quality Improvement report provided direction for health care systems on how to implement the federal standard [17]. Because the health care standard treats race and ethnicity as separate concepts, it is recommended that the question about Hispanic of Latino origin be presented first when gathering demographic information from patients. The standard has 5 minimum categories for race: (1) AI/AN, (2) Asian, (3) Black or African American, (4) Native Hawaiian or Other Pacific Islander, and (5) White. The health care standard for race data is hierarchical, with almost 900 different subcategories that could be used to describe more granular race categories, all of which are required to collapse (or “roll up”) into o1 of the 5 major categories. “Other” race is deprecated within HL7, although “unknown” and “asked but not answered” are permissible [16]. For patients who identify as multiracial, the 1997 OMB Standard and the Institute of Medicine Subcommittee both recommend allowing for the selection of more than one race rather than offering a single “multiracial” category [14,17]. However, the OMB acknowledged that allowing for multiple selections creates complications during tabulation and analysis, and the Institute of Medicine noted that “some health information technology systems are unable to support the collection and reporting of data in a ‘Select one or more’ manner” [17].

The health care standard only recommends a structure for how information about patient race and ethnicity should be stored; in practice, there are wide variations in how health care systems collect this information. Studies have documented that it is frequently missing from the patient record, and when it is collected, it is often of poor quality [18-23]. Our objective was to explore variations in how health care systems collect and report information about the race and ethnicity of their patients. To this end, we sought to assess the quality of ethnicity and race data in N3C by focusing on conformance to standard definitions, missingness, and misclassification.

## Methods

### Data Source

Although the size of the N3C Data Enclave has continued to grow, at the time of our analysis (July and August of 2021), the N3C Data Enclave contained health records from 6.5 million patients tested for COVID-19, including 2.1 million who had tested positive. The data in the Enclave are updated weekly with new information. To keep our numbers consistent, we used Release-v40-2021-07-30 to conduct analyses whenever possible; small numerical inconsistencies may appear as the result of occasions when different release versions were used.

Significant technical and regulatory hurdles were addressed to make the N3C Data Enclave available to researchers seeking insight into COVID-19. The clinical data are stored in the Observational Medical Outcomes Partnership (OMOP) Common Data Model; institutions using Accrual to Clinical Trials (ACT), National Patient-Centered Clinical Research Network (PCORnet), and TriNetX common data models have their data mapped to OMOP, while those already using OMOP have their data ingested directly. The Common Data Model Harmonization project provided syntactic mapping with conversion logic and semantic mapping to the OMOP vocabulary. N3C met with subject matter experts from source common data models and the Observational Health Data Sciences and Informatics community to finalize these mappings, which are available to the public on GitHub [12].

### Ethical Review

The protocol for this study was approved by the Institutional Review Board at Oregon Health and Science University (IRB ID STUDY00022764). This study was granted a waiver because the study design—a retrospective review of existing records—involved minimal risk. Waiver of the formal written consent process did not adversely affect the rights or welfare of the participants. This study was performed in accordance with the ethical standards as laid down in the 1964 Declaration of Helsinki and its later amendments or comparable ethical standards.

### Analyses

To quantify the variability in how health care institutions are reporting data about patient race and ethnicity to N3C, we used a multistep process that included data processing, terminology harmonization, and descriptive analyses. First, we sorted the harmonized data into “conforming” and “nonconforming” categories. We defined race and ethnicity data as “conforming” if they had been mapped to 1 of the 5 minimum categories for race congruent with the health care standard: White, Black or African American, Native Hawaiian or Other Pacific Islander, Asian, or AI/AN [14-16]. Ethnicity data were conforming if they were harmonized into 1 of the 2 standard categories for ethnicity: “Hispanic or Latino” or “Not Hispanic or Latino.” All other data—including missing data—were deemed “nonconforming.”

Next, we delved into these categories by comparing these harmonized data to their original source encodings. To make the comparison, we tabulated the original source codes, enumerating how many patients had been reported with each encoded value and how many distinct ways each category was reported. This allowed us to get a better idea of how the health care institutions were reporting the data to the N3C and to approximate how well the source institutions were adhering to the health care standard. The nonconforming data were also cross tabulated by 3 factors: patient ethnicity, the number of data partners using each code, and which data models utilized those particular encodings. For the nonconforming data, we used an inductive approach to sort the source encodings into categories. For example, values such as “Declined” were grouped with “Refused,” and “Multiple Race” was grouped with “Two or more races” and “Multiracial.”

These analyses were conducted within the N3C Data Enclave using the software tools available within the platform during July 2021 and August 2021. Additional descriptive statistics were done with Excel. Figures were developed using Lucidchart, Excel, and Keynote.

### Comparison With Other Data Sources and Repositories

To assess the external validity of the N3C race and ethnicity data, we compared race and ethnicity distributions across multiple data sources, including the 2019 American Communities Survey (ACS) demographic and housing estimates and Cerner HealthFacts (CHF) [24]. ACS data are compiled by the US Census Bureau and provide yearly updates and estimates to key demographic, economic, housing, and social data. CHF is a data warehouse that includes almost 70 million patients treated at hospitals and clinics throughout the United States between 2001 and 2017 using the Cerner EHR platform. Total unadjusted COVID-19 cases and deaths from the CDC are also included for comparison [25,26].

## Results

### Harmonized, Mapped Data

There are a total of 25 harmonized categories for race available in the N3C Data Enclave, representing mapped data contributed by 56 health care institutions. The top 10 concepts used by the N3C to describe the harmonized categories used to describe the race of the patients in the database are shown in Textbox 1. The top 3 harmonized categories—White, “No matching concept,” and Black or African American—account for 94.3% (6,140,139/6,513,464) of the data. No patients with race of AI/AN were found; at the request of the NIH, the health records of AI/AN patients were intentionally obscured during ingestion (see Discussion) [27].

**Top 10 harmonized concepts used by the National COVID Cohort Collaborative (N3C) to describe the race of patients in the database.**

1. White
2. No matching concept
3. Black or African American
4. Asian
5. Null
6. Unknown
7. Other
8. Other race
9. Black
10. No information

**Figure 1:**
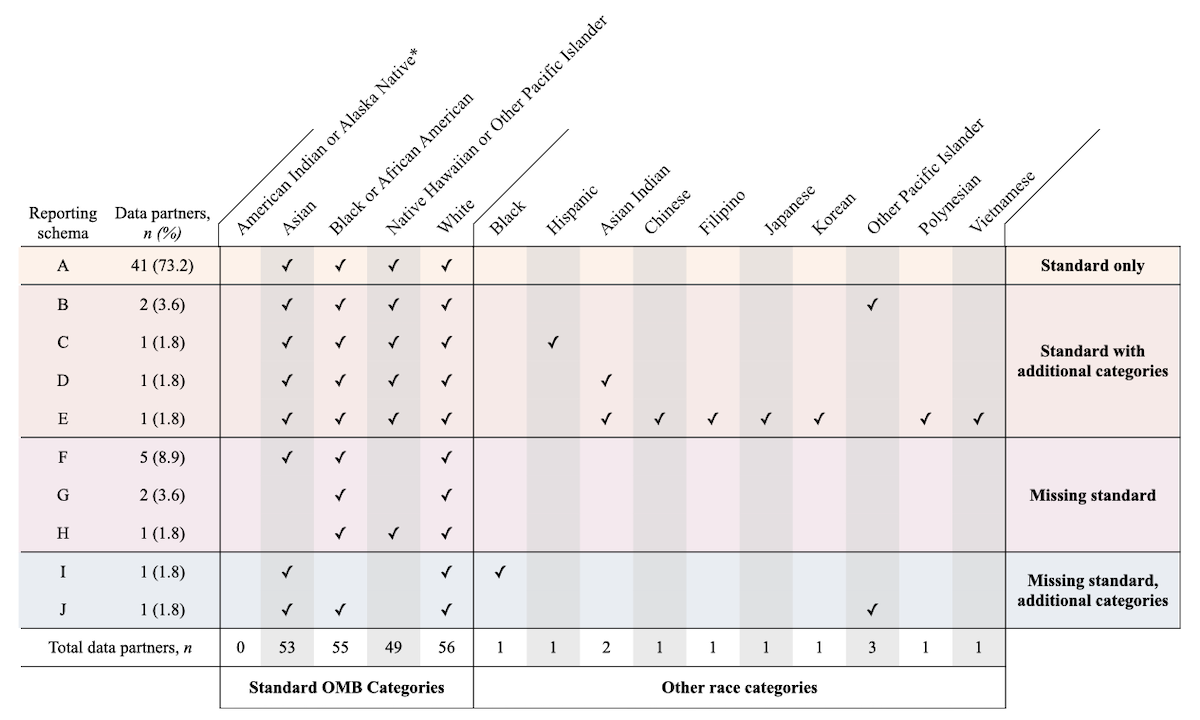
Race data reporting schema by contributing sites. Although data partners did contribute data on American Indian or Alaska Native patients, as noted elsewhere, these data were intentionally obscured. OMB: Office of Management and Budget.

Following ingestion and mapping to standard OMOP race categories, we identified 10 different reporting schema (combinations of reported race categories) among contributing institutions as shown in Figure 1. Of the 56 data partners, 41 (73%) had harmonized patient race data that adhered to the standard OMB race categories other than AI/AN, as noted in the previous paragraph. Of the data partners, 5 had harmonized data that included all the standard race categories, plus some additional categories such as Filipino or Korean that had not been correctly rolled up into main categories for tabulation purposes (both are subcategories of Asian). Data from 10 contributing institutions (10/56, 18%) omitted at least one of the standard race categories other than AI/AN. The only OMB race category present for all data partners was White. Ethnicity was as a separate field present for 51 (51/56, 91%) of the data partners.

### Conforming Data

**Figure 2:**
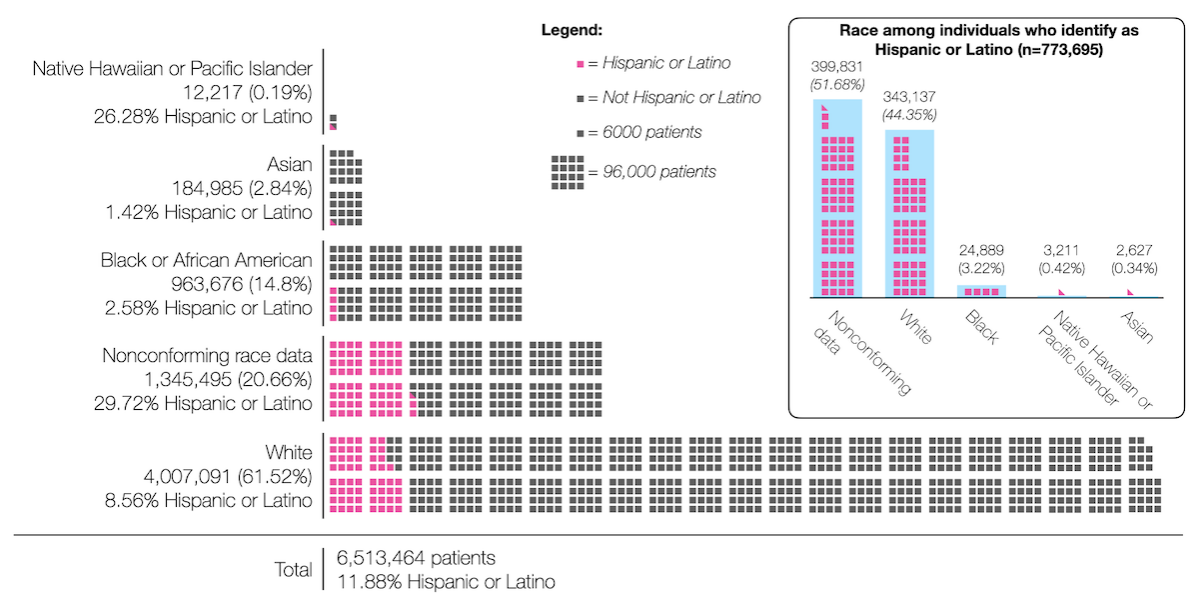
Race and ethnicity data in the National COVID Cohort Collaborative (N3C) after harmonization.

Of the data about race, 79.3% (5,167,969/6,513,464) had been harmonized to 1 the 5 main categories recommended in the health care standard. Examining the harmonized data that does conform to the standard, Figure 2 illustrates the racial and ethnic makeup of patients in N3C. The source data showed that White race was originally reported to the N3C by health care institutions a total of 21 different ways, most commonly using the PCORnet code 05 (1,442,961/4,007,091, 36.0% of all White patients). “Jewish” was the only granular category available in the source data for patients whose race had been mapped to White, and 141 of the patients whose race had been mapped to White had Jewish ancestry recorded in the source data.

The most common code found in the source data to report Black or African American patients was PCORnet’s encoding 03 (411,537/963,676, 42.7%). Although the source data contained 24 different encodings for this group, there were no granular subcategories of Black or African American available in either the source or the mapped data.

The source data for patients whose race had been harmonized to Asian showed 22 distinct encodings, most commonly using the PCORnet code 02 (77,426/184,985, 41.9% of all Asian patients). Source data revealed 6 more granular race categories had been rolled up into Asian during the harmonization process; these more granular data represented 546 patients. The most common of these granular subcategories was Asian Indian (n=388). However, in the harmonized data, there were 1534 additional Asian Indian patients who were not rolled up into the Asian category.

Native Hawaiian or Other Pacific Islander is the smallest of the standardized racial categories found in the Enclave, and the data were initially reported using 24 different encodings prior to harmonization. In the source data, we found 2 granular subcategories that had been rolled up into Native Hawaiian or Other Pacific Islander: Guamanian/Chamorro and Polynesian. This group contained the largest proportion of people who also identified as Hispanic or Latino.

Overall, 83.8% (5,456,162/6,513,464) of the data about ethnicity conformed to the standard. There was a total of 26 different encodings to represent Hispanic or Latino ethnicity in the source data, including 7 granular subcategories such as Puerto Rican, Mexican, and South American.

### Nonconforming Data

**Figure 3:**
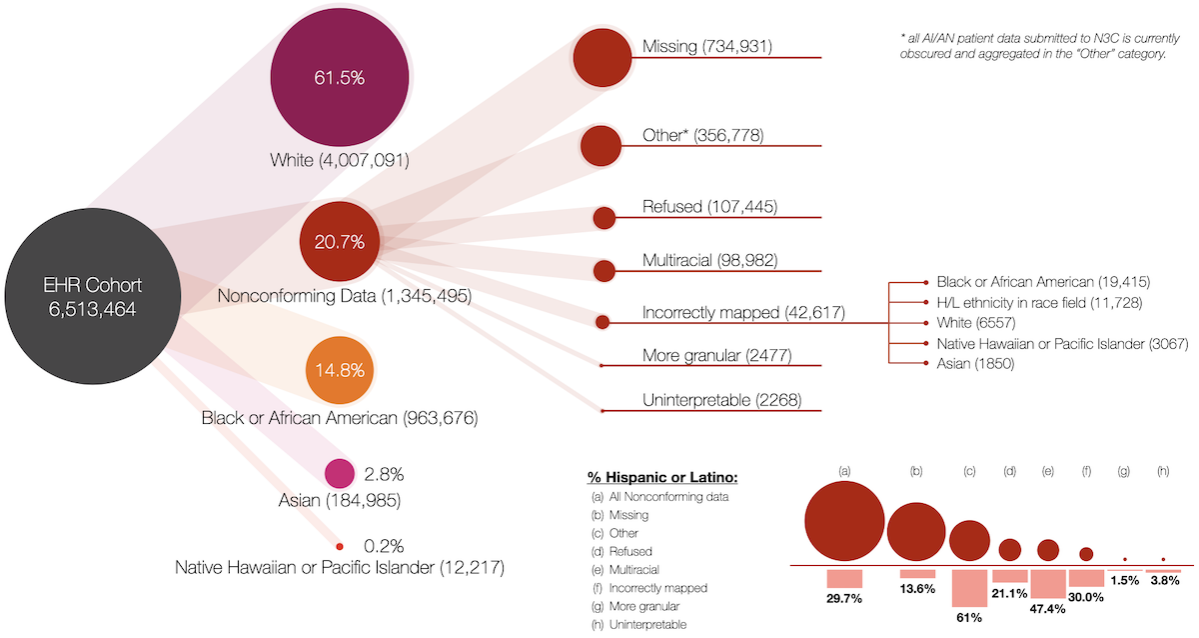
Weighted tree diagram of nonconforming race data. AI/AN: American Indian or Alaska Native; EHR: electronic health record; H/L: Hispanic/Latino; N3C: National COVID Cohort Collaborative.

About 20.7% (1,345,495/6,513,464) of the data in the N3C had not been harmonized to one of the 5 primary race categories described in the health care standard. As shown in Figure 3, nonconforming data could be divided into 7 categories.

#### Missing

Incompleteness was the most common reason for race data to be nonconforming, and source data showed that 11.3% (734,931/6,513,464) of all patients in the N3C Data Enclave were marked as missing race data. Of the contributing health care institutions, 31 reported missing data in 29 distinct ways; most often, a zero was recorded to indicate that the data were incomplete (n=348,057). Of the patients missing data about race, 13.6% (99,853/734,931) were noted as being Hispanic or Latino in the ethnicity column.

#### Other

The second largest category of nonconforming race data was patients labeled by health care systems as “Other” race. The majority of these patients (217,476/356,778, 61.0%) was recorded as being of Hispanic or Latino ethnicity. Currently, all AI/AN patient data submitted to N3C are obscured and aggregated in the “Other” category [28]. This transformation has thus far rendered data from this cohort unavailable to researchers.

#### Refused

Patients who declined to answer questions about race represented 8.0% (107,445/1,345,495) of the nonconforming data. However, examining the data about ethnicity showed that 21.1% (22,683/107,445) of these patients were Hispanic or Latino.

#### Multiracial

Multiracial patients represented 7.4% (98,979/1,345,495) of the nonconforming data and 1.5% (98,979/6,513,464) of all the patients in the N3C Data Enclave. Of the 257 different codes used by systems to represent race in the nonconforming data, 119 of them were distinct codes used to represent multiracial patients. Much of the variety was due to some systems allowing patients to select multiple races, which was then reported as several selections in a single column. Although only 3.8% (3764/98,979) of all multiracial patients actually had more than one race recorded, this 3.8% represented 101 different combinations of codes. The most common of these were combinations of White and Black or African American (n=1563).

#### Misclassified

Examining the source data revealed that 3.1% (42,617/1,345,495) of the nonconforming race data, 0.6% (42,617/6,513,464) of all data in the N3C, were not mapped to the appropriate standard race concepts. Although only 14.8% (963,676/6,513,464) of the patients in the Enclave are Black or African American, source data showed that 45.6% (19,415/42,617) of these misclassified patients should have had their race mapped to Black or African American. The next largest group of misclassified patients was those whose source institutions had recorded Hispanic or Latino ethnicity in the race field (n=11,728)—the N3C Data Enclave treats Hispanic or Latino separately from race. Confusingly, 19.3% (2258/11,728) of the patients whose race was reported as Hispanic or Latino were labeled as Not Hispanic or Latino in the ethnicity field. Patients identified as White represented 15.9% (6557/42,617) of the misclassified nonconforming data; 7.4% (3067/42,617) of these misclassified patients were Native Hawaiian or Other Pacific Islander, and 4.5% (1850/42,617) were Asian.

#### Uninterpretable

For 2268 patients, the source institution had provided a code such as “@” that did not conform to those recognized by any of the known data models. There were 2 encodings we were unable to decipher, both of which came from institutions using the TriNetX Common Data Model.

#### More Granular

Finally, 2477 patients did not map to 1 of the 5 categories because they had been labeled with a granular racial subcategory that had not been rolled up into 1 of the 5 main race categories. Nine racial subgroups are available in the nonconforming data in the N3C; 6 of these (Asian Indian, Filipino, Chinese, Korean, Vietnamese, and Japanese) should have been rolled up into the larger category of “Asian.” The most widely reported subcategory we found in the nonconforming data was “Asian Indian” (n=1534). These granular data all came from health care systems using the OMOP Common Data Model.

For patients with Hispanic or Latino ethnicity, “nonconforming” was the single largest racial category (399,831/773,695, 51.7%). One data partner mapped 2533 patients whose race had originally been recorded as Hispanic or Latino to a racial category that was subsequently labelled only as “non-White.”

### Variations by Common Data Model

Four common data models are used by the health care institutions contributing data to the N3C Data Enclave: OMOP, PCORnet, TriNetX, and ACT. Some OMOP sites also included data in the PEDSnet common data model, which is an extension of OMOP that includes pediatric-specific data fields and standards such as age-normalized anthropometrics [29]. The Enclave itself uses the OMOP model, and non-OMOP contributing institutions preprocess their data so they can be harmonized to the OMOP model. When stratifying the patient data by the data model used by their health care institution, we found that data about patient race from TriNetX had the best conformance; 86.2% (711,075/825,001) of the TriNetX data conformed to 1 of the 5 main categories. Only 66.2% (64,242/97,097) of the race data from OMOP PEDSnet, on the other hand, achieved conformance. We found that, depending on the data model, the conformance of data about ethnicity varied more widely than the race data; although 93.1% (2,146,229/2,305,731) of data from health care institutions using PCORnet’s data model conform to the standard for reporting patient ethnicity, only 50.8% (271,304/534,179) of ethnicity data from institutions using the ACT model were adherent to the standard.

### Comparison With Other Data Sources

**Figure 4:**
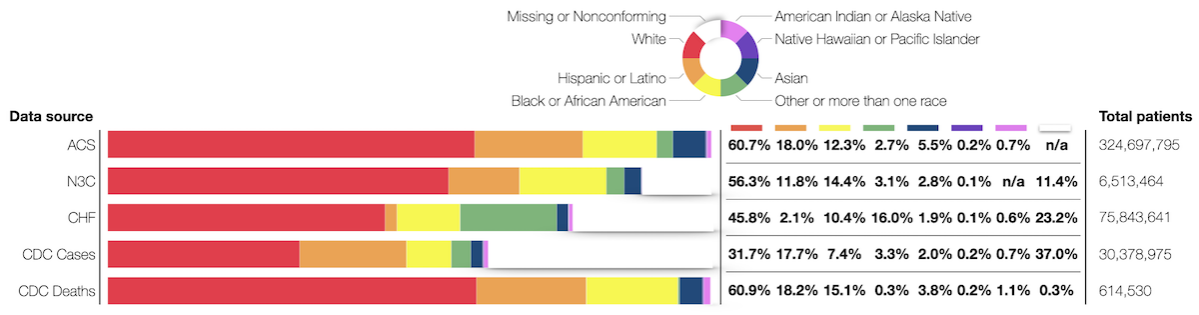
Comparisons of race and ethnicity data across data sets. Data from American Indian or Alaska Native patients in the National COVID Cohort Collaborative (N3C) are labeled “not applicable” because these data were obscured until the completion of the Tribal Consultation. ACS: American Communities Survey; CDC: Centers for Disease Control and Prevention; CHF: Cerner HealthFacts.

Figure 4 shows how the distribution of race and ethnicity data in N3C compares with the United States overall (ie, the ACS) and 1 other EHR-based data repository, CHF. N3C, similar to CHF, has fewer Hispanic or Latino and Asian patients than the ACS but comparable rates for other groups. This is likely related to both the types of institutions that contribute data (and the patients they serve) as well as the large amount of missing or nonconforming data in both data sets.

## Discussion

### Principal Findings

Our analyses of the N3C Data Enclave revealed a number of facts that are important for researchers to consider when drawing conclusions based on these data. First, “no matching concept” was the second largest harmonized racial group in the N3C. A substantial portion of the records (20.7%) were in some way nonconforming, including 11.7% of all records that were missing race or ethnicity data (missing data were considered a subcategory of nonconformance in this study). Second, while data collection at the point of care needs improvement, there are also opportunities to improve the quality of these data at various points in the data pipeline. Finally, the impact of these data quality issues was not equal across all races and ethnicities. The magnitude and type of nonconformance varied across race and ethnicity, with patients of color and vulnerable communities overrepresented in the misclassified data and nonconforming data.

### Implications for COVID-19 Research

The fact that the data were not randomly nonconforming means there is potential to introduce bias in analyses and conclusions drawn from these data. Because data in categories such as “other” or “missing” are often discarded by data users, any patients in those categories are at risk of being inadvertently excluded from research. Data we refer to as nonconforming included several categories that should have been either mapped or rolled up into a main category. For example, we found that the harmonized data included 18,885 patients who had been categorized as “Black” instead of being mapped into the standardized “Black or African American” category. This indicates a significant amount of heterogeneity in the data about race, an issue that may fracture research cohorts and create noise in the data. This could cause problems if data users conducting queries on the N3C database pull the information from one group and inadvertently omit the other. At best, this can be a rate-limiting factor for researchers who must then spend extra time harmonizing the data and doing the mapping themselves rather than studying COVID-19.

It is, however, necessary to put these findings in context. The problems with the data about race and ethnicity are not exclusive to the N3C; indeed, a report from the CDC entitled “Addressing Gaps in Public Health Reporting of Race and Ethnicity for COVID-19” documented the same issue with public health data [30]. Compared with the CDC data on COVID-19 cases, the N3C system has significantly less missingness at 11%, compared with 24% in the CDC COVID-19 case data set. Of the data, 79% conform to CDC standard racial categories (higher than the 64% in the CHF data set), making this repository useful for COVID-19 health disparities research. Moreover, because the N3C Data Enclave gives access to race and ethnicity source values, we were able to assess misclassification and can update the racial and ethnic categorization of patients for research purposes. Though granular data represent a small portion of the overall data, they can be used for small-scale projects analyzing differences within racial categories. Given that this data set has representation from all regions in the United States [8], it can be used to validate against the CDC COVID-19 positivity rates by race.

### Mismappings

Although an exhaustive assessment of the causes of mismapping is not feasible given the various mapping and transformation steps that occur upstream of the N3C Data Enclave, many occur during site-level data entry and processing. For example, contributing sites employ standard scripts to map EHR data to common data models. When data preparation is automated using such scripts, patients who have been assigned deprecated codes at the point of care may ultimately be harmonized to “No matching concept.” Misclassification of patients might also occur if multiple values have been entered into a single field, as when more than one race has been selected or when sites use the “single question” format when gathering demographic information. We hope to utilize the results of this analysis to add additional coding to these scripts to prevent misclassification and to identify ways to correct the race and ethnicity data postingestion.

### “Hispanic or Latino,” Race, and Ethnicity

Our finding that Hispanic or Latino patients are overrepresented in the nonconforming race data may reflect that the 2-concept system (ie, recording “race” and “ethnicity” separately) continues to be a source of variability. Although the health care standard recommends that self-reported race and ethnicity be collected as separate concepts, some health care systems combine them and offer “Hispanic or Latino” as a possible selection under Race. The current PCORnet common data model specification recommends mapping data from patients whose race has been recorded as Hispanic or Latino to “Other,” which explains some of our finding that 61% of the data harmonized to the “Other” category come from Hispanic or Latino patients [31]. During the 2010 Census, the US Census Bureau tested a combined race-ethnicity question and found that including Hispanic origin as a racial category dramatically reduced both the item nonresponse rate and the selection of “some other race.” The results of the Census testing suggest that the issues with Hispanic/Latino data are, at least in part, attributable to the 2-question structure [32]. However, it should be noted that Hispanic or Latino patients were also overrepresented in other categories of nonconforming data, such as “Refused” (21.1% Hispanic or Latino) and “Multiracial” (47.4% Hispanic or Latino). This suggests that the heterogeneity in the data from Hispanic/Latino patients may also be a result of the difficulty people have selecting from standardized categories that they feel do not adequately represent them.

### Obscured Data From American Indian or Alaska Native Patients

The lack of accessible data on AI/AN populations is a limitation of the data set. The Urban Indian Health Institute has stated that “current standard data collection practices by many federal, state, and local entities effectively omit or misclassify AI/AN populations, both urban and rural. This is particularly concerning in the midst of the COVID-19 pandemic as these current standards of practice are resulting in a gross undercount of the impact COVID-19 has on Native people” [33]. A number of federal laws, treaties, and executive orders has established the sovereignty of Tribes and Tribal Nations over their data and the power to regulate research, although the gap between *recognition* of those rights and the *assertion* of those rights remains wide [34-36]. A legacy of harm, medical maltreatment, and research misconduct has engendered mistrust between the Tribal and clinical research communities [36]. To begin to address these issues, Tribal leaders, scholars, and advocates have established protocols and institutions to ensure human protections for research involving the AI/AN community [37]. In 2010, the US Department of Health and Human Services established a formal Tribal Consultation Policy to create a mechanism for collaboration at the federal level [38].

In December of 2021, the NCATS formally initiated a Tribal Consultation about the N3C Data Enclave. The NCATS Framing Letter, “NIH Tribal Consultation on the National COVID Cohort Collaborative (N3C),” states, “Ideally, NIH would have sought Tribal Consultation before the start of this program. However, given other COVID-related Consultations and urgency of the pandemic, NCATS decided to obscure AI/AN data until consultation could occur. During the consultation, the NIH will seek input on whether and how to make AI/AN data available through N3C” [28]. The N3C Tribal Consultation took place on February 11, 2022, and as of this writing, the testimony of the Tribal Leadership is being collected. NCATS expects to implement their recommendations by summer 2022.

It is important to note that our analysis is not an endorsement of the standard developed by the OMB and implemented by federal agencies but rather a description of how health care institutions around the United States have been implementing it. Indeed, our position is that deviations from the standard are a signal of the manner in which such categories are both arbitrary and reductionist. Our perspective is that race and ethnicity are not biological categorizations; instead, they should be viewed as social constructs that are highly context-dependent and tied to existing power dynamics. The US Census Bureau stresses this point, stating that these categories “generally reflect a social definition of race recognized in this country and not an attempt to define race biologically, anthropologically, or genetically” [39]. As variables in clinical research, the utility of race and ethnicity is that they can be used as highly imperfect proxies for the complex systemic factors (eg, racism, colonialism, socioeconomic barriers to health care delivery systems) that drive and perpetuate inequities [40,41].

### Limitations

Finally, it should be noted that data partners, as the contributing health care institutions are referred to in the N3C, were provided with anonymity as a consideration for contributing data. This means that the provenance of these data is limited, so we do not know how they were initially collected. Finally, because most of the contributing health care institutions are recipients of a Center for Translational Science Award with an established relationship with the NCATS, it is likely that academic medical centers are overrepresented.

### Conclusion

Twenty-eight years after Congress mandated the inclusion of racial and ethnic minority groups in federally funded clinical research with the NIH Revitalization Act [42], the ongoing lack of racially and ethnically diverse cohorts remains a challenge to improving equity in research and health care [43]. Because the COVID-19 pandemic disproportionately impacts communities of color by exacerbating existing health inequities, the accurate identification of these cohorts within N3C is crucial to identifying, understanding, and ultimately addressing these disparities. Data problems arise for many reasons, but primary among them is the discrepancy between how institutions conceptualize race and ethnicity and the far more varied ways people identify themselves [44]. The complex history of racial identification in the United States has resulted in shifting concepts of race and ethnicity [45]. Self-identified race and ethnicity are often dependent on physical attributes that, although heritable, correlate poorly with genetic similarity or ancestry. Nevertheless, race and ethnicity are well-established predictors of health outcomes and access to care. However, a multitude of factors that are both correlated with and are independent of race and ethnicity may affect group differences in health and health care. Race and ethnicity are only one of many elements considered to be social determinants of health—nonmedical factors that influence health outcomes and are known to have a significant relationship with these disparities [46,47]. Teasing out which factors influence health outcomes is challenging [48], and issues with data quality and inappropriate or poorly applied standards around race and ethnicity can greatly lessen our understanding of health disparities [17].

Though there are some limitations to the racial representation in this data set, it nevertheless remains a unique resource for COVID-19 research on racial disparities. COVID-19 has served to emphasize the deadliness of these disparities and has made social conditions far worse for many Black, Hispanic, and American Indian persons living in the United States. However, these inequities are not immutable. The COVID-19 pandemic provides an opportunity for clinicians, health systems, scientists, and policy makers to address social disparities and thereby improve the health and well-being of all persons in the United States for both known and future illnesses.

Databases such as N3C spur discovery by collecting and centralizing clinical data, making national, centralized data sets available to researchers. Although intended to increase the accessibility of data, governance can paradoxically create further restrictions. Centralization efforts require that data be transformed numerous times, and differences in how race and ethnicity are conceptualized, documented, and encoded by health care institutions affect the quality of the harmonized data. Across the full data life cycle, more transparency about these numerous decisions is critical if researchers are to make accurate inferences from analyses. Careful and systematic analyses are important to better guide clinical care and public policy but also to inform iterative improvement of collection and harmonization across the EHR data life cycle.

## Abbreviations

ACS: American Communities Survey
ACT: Accrual to Clinical Trials
AI/AN: American Indian or Alaska Native
CDC: Centers for Disease Control and Prevention
CHF: Cerner HealthFacts
EHR: electronic health record
FHIR: Fast Healthcare Interoperability Resources
HL7: Health Level Seven International
N3C: National COVID Cohort Collaborative
NCATS: National Center for Advancing Translational Sciences
NIH: National Institutes of Health
OMB: Office of Management and Budget
OMOP: Observational Medical Outcomes Partnership
PCORnet: National Patient-Centered Clinical Research Network
